# Measuring geographical accessibility to palliative and end of life (PEoLC) related facilities: a comparative study in an area with well-developed specialist palliative care (SPC) provision

**DOI:** 10.1186/s12904-017-0185-0

**Published:** 2017-01-26

**Authors:** Clare Pearson, Julia Verne, Claudia Wells, Giovanna M. Polato, Irene J Higginson, Wei Gao

**Affiliations:** 1 King’s College London, Department of Palliative Care, Policy and Rehabilitation, Cicely Saunders Institute, Bessemer Road, London, SE5 9PJ UK; 2 Office for National Statistics, Life Events and Population Sources Division, Newport, NP10 8XG UK; 3 Public Health England, National End of Life Care Intelligence Network, 2 Rivergate, Temple Quay, Bristol, BS1 6EH UK; 4 Care Quality Commission, 151 Buckingham Palace Road, London, SW1W 9SZ UK

**Keywords:** Palliative care, Geographical accessibility, Methods, Health services, Accessibility

## Abstract

**Background:**

Geographical accessibility is important in accessing healthcare services. Measuring it has evolved alongside technological and data analysis advances. High correlations between different methods have been detected, but no comparisons exist in the context of palliative and end of life care (PEoLC) studies. To assess how geographical accessibility can affect PEoLC, selection of an appropriate method to capture it is crucial.

We therefore aimed to compare methods of measuring geographical accessibility of decedents to PEoLC-related facilities in South London, an area with well-developed SPC provision.

**Methods:**

Individual-level death registration data in 2012 (*n* = 18,165), from the Office for National Statistics (ONS) were linked to area-level PEoLC-related facilities from various sources. Simple and more complex measures of geographical accessibility were calculated using the residential postcodes of the decedents and postcodes of the nearest hospital, care home and hospice. Distance measures (straight-line, travel network) and travel times along the road network were compared using geographic information system (GIS) mapping and correlation analysis (Spearman rho).

**Results:**

Borough-level maps demonstrate similarities in geographical accessibility measures. Strong positive correlation exist between straight-line and travel distances to the nearest hospital (rho = 0.97), care home (rho = 0.94) and hospice (rho = 0.99). Travel times were also highly correlated with distance measures to the nearest hospital (rho range = 0.84–0.88), care home (rho = 0.88–0.95) and hospice (rho = 0.93–0.95). All correlations were significant at *p* < 0.001 level.

**Conclusions:**

Distance-based and travel-time measures of geographical accessibility to PEoLC-related facilities in South London are similar, suggesting the choice of measure can be based on the ease of calculation.

**Electronic supplementary material:**

The online version of this article (doi:10.1186/s12904-017-0185-0) contains supplementary material, which is available to authorized users.

## Background

Conceptually, accessibility can be difficult to define. Geographical accessibility has long been used as one dimension of overall accessibility (in addition to availability, affordability and acceptability) [1]. Being physically able to access healthcare services (proximity and/or being able to travel) is an important human right and equity of access to healthcare is optimal, including palliative and end of life care (PEoLC) related services. Geographical accessibility to healthcare has been investigated in a wide range of health services research, including accessing primary healthcare facilities [2, 3] examining colorectal cancer survival [4] and renal replacement therapy services [5].

Increasingly sophisticated methods have been utilised to calculate geographical accessibility to services as technical advancements in data management and more advanced Geographic Information Systems (GIS) techniques have developed. Each method requires assumptions about populations and/or services. Simple straight-line distance calculations are easier to compute, interpret and compare, but assume that someone travels to their nearest facility and ignores topographical features such as rivers and hills which could interfere with a straight-line journey. Travel distances and times are more suitable where road networks deviate from straight lines but assume access to a private car and that constant traffic flows are uninterrupted by roadworks, accidents, congestion or adverse meteorological conditions. These simpler methods ignore the size and capacity of the nearest healthcare provider or other factors possibly influencing access decisions (e.g. quality, reputation). More complex models (gravity and floating catchment methods) can take provider factors and boundaries into account. However, they require complex computation and more data processing capacity and produce results that may be difficult to interpret and compare.

Previous comparisons of methods to calculate geographical accessibility to healthcare services detected high correlations between straight-line distances and drive time to A & E services in Wales [6], to hospitals in North England [7], to hospitals and GPs in South West England [8, 9]. In North America similarly high correlations were found between straight-line and driving distances to hospitals [10], and between straight-line distances and travel times [11], particularly in metropolitan areas [12]. These findings suggest that road distances only marginally improved predictive accuracy over using straight-line distances.

Considerations of people utilising healthcare facilities towards the end of life may include additional specific needs, such as family and friends to be able to access facilities readily. This information is valuable to policy-makers and care providers to optimise current service provision and organisation, which may lead to improved efficiency and reduced inequality in end of life care. The information is also useful for researchers to gain a better understanding of the mechanism underlying care inequality. In the palliative and end of life care field, geographical accessibility to palliative care services has been examined in Canada, where access to palliative care for rural and hard to reach communities [13] and travel time analysis around specialist palliative care (SPC) were investigated [14, 15]. In Australia straight-line distances between place of residence and SPC provider were examined [16]. USA-based studies have examined ratios of services by geographical area [17, 18], community palliative care services in rural areas [19], children’s hospices in Tennessee [20] and travel times to hospices from population (census) centroids [21]. In England and Wales, geographical variation in access to inpatient hospices using driving times between small areas (Lower Super Output Area (LSOA), which each contain approximately 1500 people) and inpatient hospice locations was described [22] alongside straight-line distances. However, the two methods were not statistically compared.

Healthcare services in a local area was identified as one of many potential factors affecting place of death [23], however, this relationship has not been evaluated systematically before, and an important component of this is determining the method of calculating geographical accessibility. This decision should be based on the most practical, pragmatic and available methods, as should future studies in the palliative care field involving geographical accessibility. No study to date has compared methods for calculating geographical accessibility within palliative care research to determine which method can be reliably used by service providers, commissioners and other interested parties.

Therefore we aimed to compare methods of measuring geographical accessibility for people who died during 2012 in South London to their nearest PEoLC-related healthcare services.

## Methods

### Setting/Design

This was a population-based study in South London with data from 2012. The boroughs of London located south of the river Thames include inner (Greenwich, Lambeth, Lewisham, Southwark and Wandsworth) and more suburban outer-London environments (Bexley, Bromley, Croydon, Kingston upon Thames, Merton, Richmond upon Thames, Sutton). The socio-economically and ethnically diverse boroughs of South London are almost entirely designated as urban metropolitan areas. South London also has strong local networks of SPC services.

### Datasets

#### Individual level data

The Office for National Statistics (ONS) individual level death registration dataset includes postcodes of usual place of residence and the place of death for each decedent in England. Ethical approval was granted by the King’s College London Research Ethics committee for secondary analysis of the death registration data (Reference number: BDM/14/15-5). All adult deaths (> = 25 years) in South London boroughs from the year 2012 (excluding those dying from external causes - ICD-10: S00-Y98), were included in the analysis. We chose to focus on non-external causes only, as people who are dying from these causes could potentially benefit from palliative and end of life care. The 2012 data was chosen as the most recent year of cleaned and checked death registration data available at the start of the analysis. The full postcode of usual residence was used for geocoding.

#### Area-level data

Borough-level ONS mid-year 2012 population estimates were used to calculate ratios. Locations of the healthcare facilities were obtained from various open-access sources. The Hospital data (2012) were provided by the Health and Social Care Information Centre (HSCIC); Care home data for 2012 were supplied by the Care Quality Commission (CQC) and Hospice data (2012) were provided by the National Council for Palliative Care (NCPC) and the London Cancer Alliance (LCA). Each dataset was checked and cleaned and facilities were geocoded from their full postcode. In the hospital dataset, facilities which are solely private, psychiatric or community were excluded. Children’s hospices were excluded from the hospice dataset. Care homes with and without nursing were included. Healthcare facilities located outside of the geographical boundaries of South London boroughs but sharing the same postcode prefix as locations within South London boundaries were retained for the distance calculations. This was to ensure that the nearest facilities to residents near the outer borders of boroughs were captured; the nearest facility may be located just outside of the South London boundary and patients/people are not always restricted to attend healthcare facilities within their borough.

### Measures of geographical accessibility

For each decedent, the following measures were calculated for three categories of healthcare facilities (hospitals, care homes and hospices):

1. Ratios of facilities by 100,000 people by borough
2. Straight-line Euclidean distances between postcode of residence and the nearest facility (“Crow flies”)
3. Travel distances along the road network between postcode of residence and nearest facility
4. Travel times along the road network between postcode of residence and nearest facility

The Ordnance Survey Integrated Transport Network database was used as the road network dataset for the travel distance and travel time analysis. ArcGIS (v 10.1 – ESRI) was used to calculate the distance and travel time measures by importing the datasets, converting them to shapefiles and using the geoprocessing functions of the software.

### Statistical analysis

The ONS death registration dataset was checked and cleaned. Each accessibility score was calculated for every decedent to the three categories of healthcare facility and merged using a pseudonymised personal identifier, so that all decedents had accessibility scores for each method for each facility type (hospitals, care homes and hospices). Descriptive analysis of the population and the accessibility measures was undertaken. Maps at borough level were produced to illustrate visual differences or similarities between methods.

Accessibility measures 2–4 were compared using Spearman's rank correlation analysis, this was based on the non-normal distribution of the accessibility variables and categorical nature of the buffers.

Accessibility results were summarised and mapped (in quintiles) by South London borough; small numbers in some categories and the resultant instability meant that maps at a smaller geographical scale were not possible to produce.

STATA (v13) was used for the statistical analysis.

## Results

There were 18,165 non-external causes of deaths in 2012 in South London. The median age of the decedents was 81 years (SD: 13, range 25–108), 52% were women with 35% married, 39% widowed and 14% single. 30% had cancer as the underlying cause of death, 20% cardiovascular diseases, 7% cerebrovascular diseases, 9% Dementia & Alzheimer’s Disease and 6% Chronic Obstructive Pulmonary disease. 62% resided in the outer South London boroughs.

The median distance to the nearest hospital using the straight-line distance method was 2533.4 m, the travel distance medians were unsurprisingly longer (3310 m). The median distances to the nearest care home and hospices were shorter and longer respectively, reflecting the number of each within South London included in the analysis (hospitals - *n* = 26; care homes – *n* = 1076; hospices – *n* = 5). The summary statistics for each method of accessibility to individual healthcare facility are provided in Additional file 1: Table S1.

**Fig. 1:**
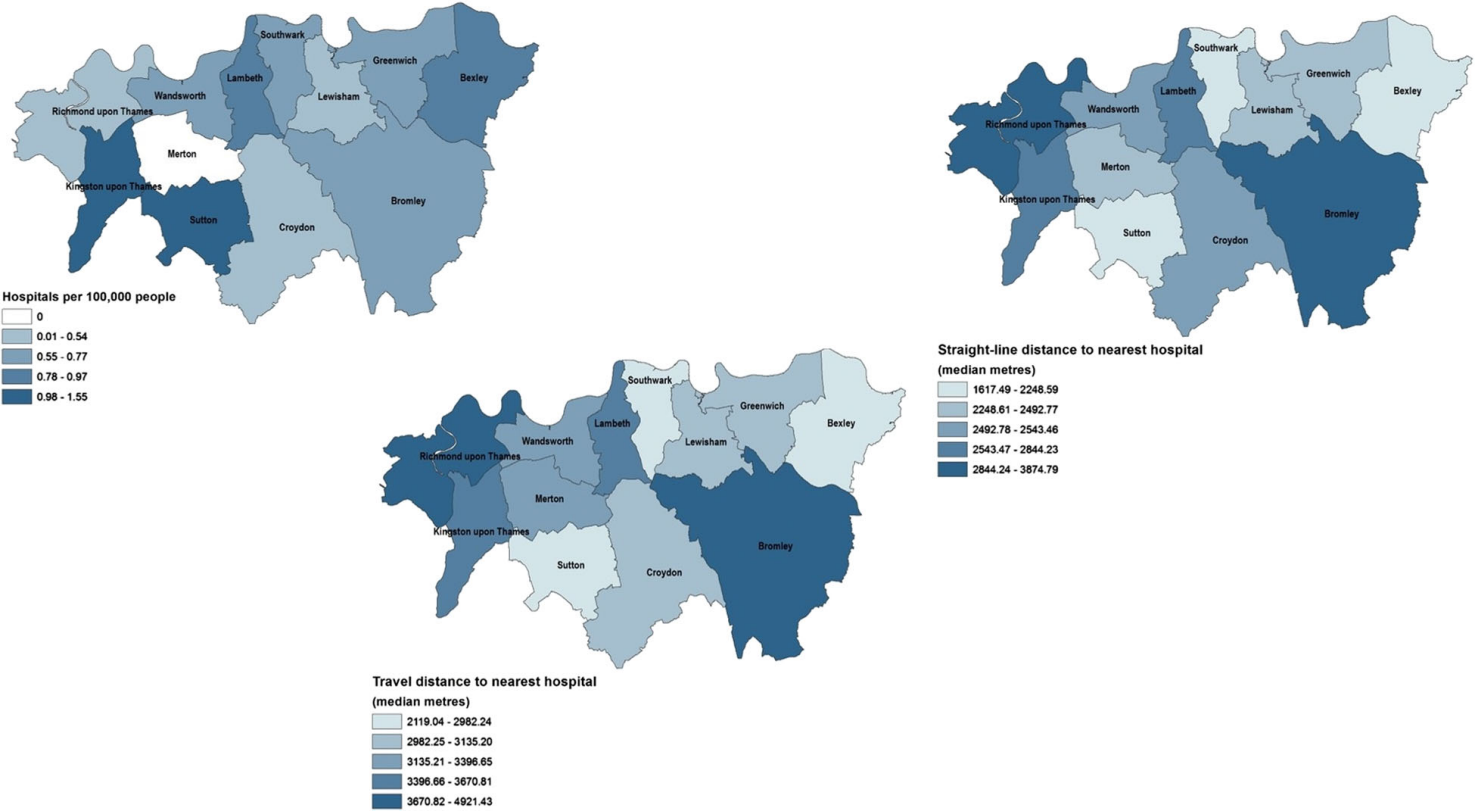
Accessibility measures to the nearest hospital, by borough, 2012. The digital boundary file contains National Statistics data © Crown copyright and database right [2012] and contains Ordnance Survey data © Crown copyright and database right [2012]

**Fig. 2:**
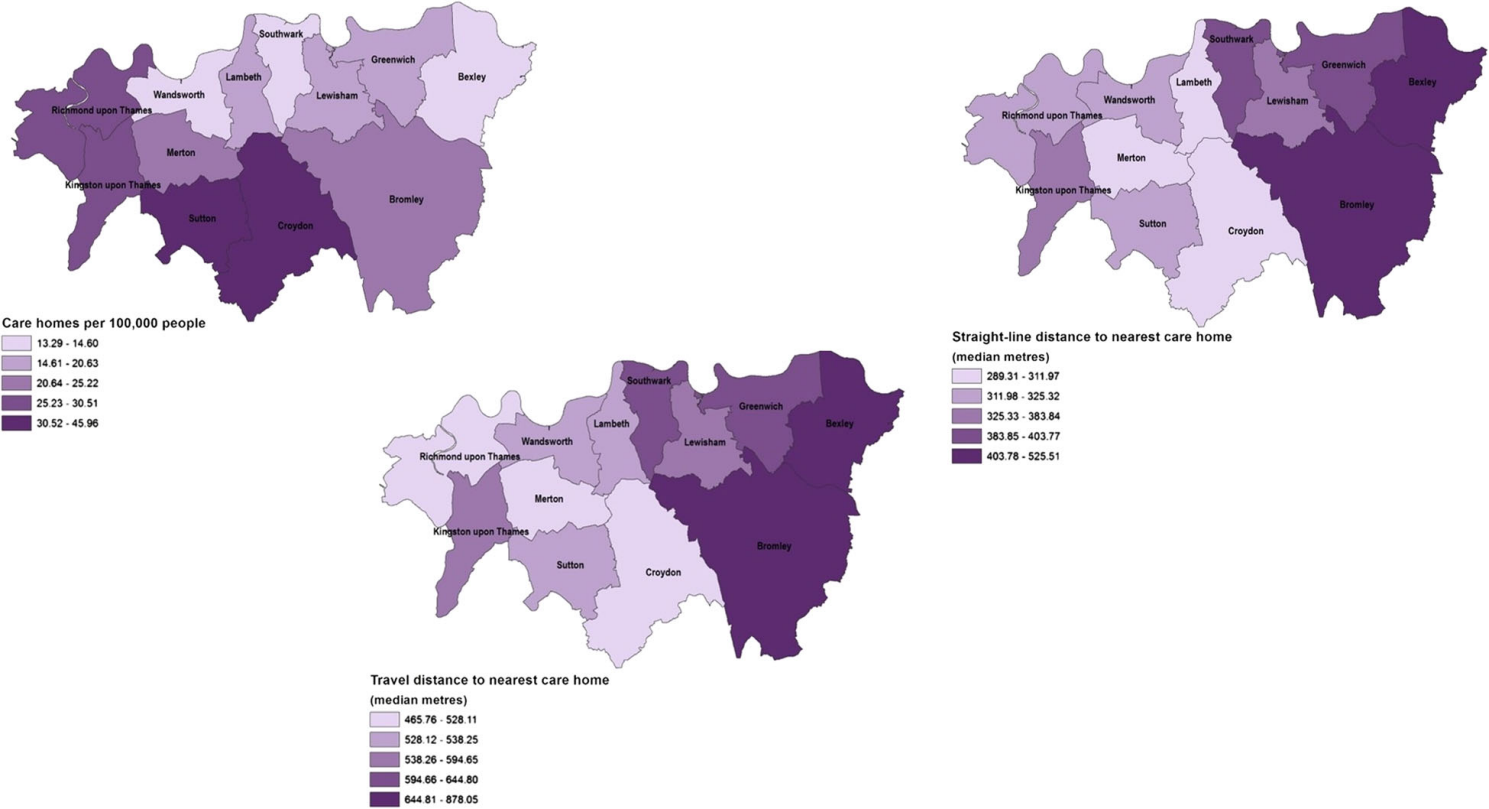
Accessibility measures to the nearest care home, by borough, 2012. The digital boundary file contains National Statistics data © Crown copyright and database right [2012] and contains Ordnance Survey data © Crown copyright and database right [2012]

**Fig. 3:**
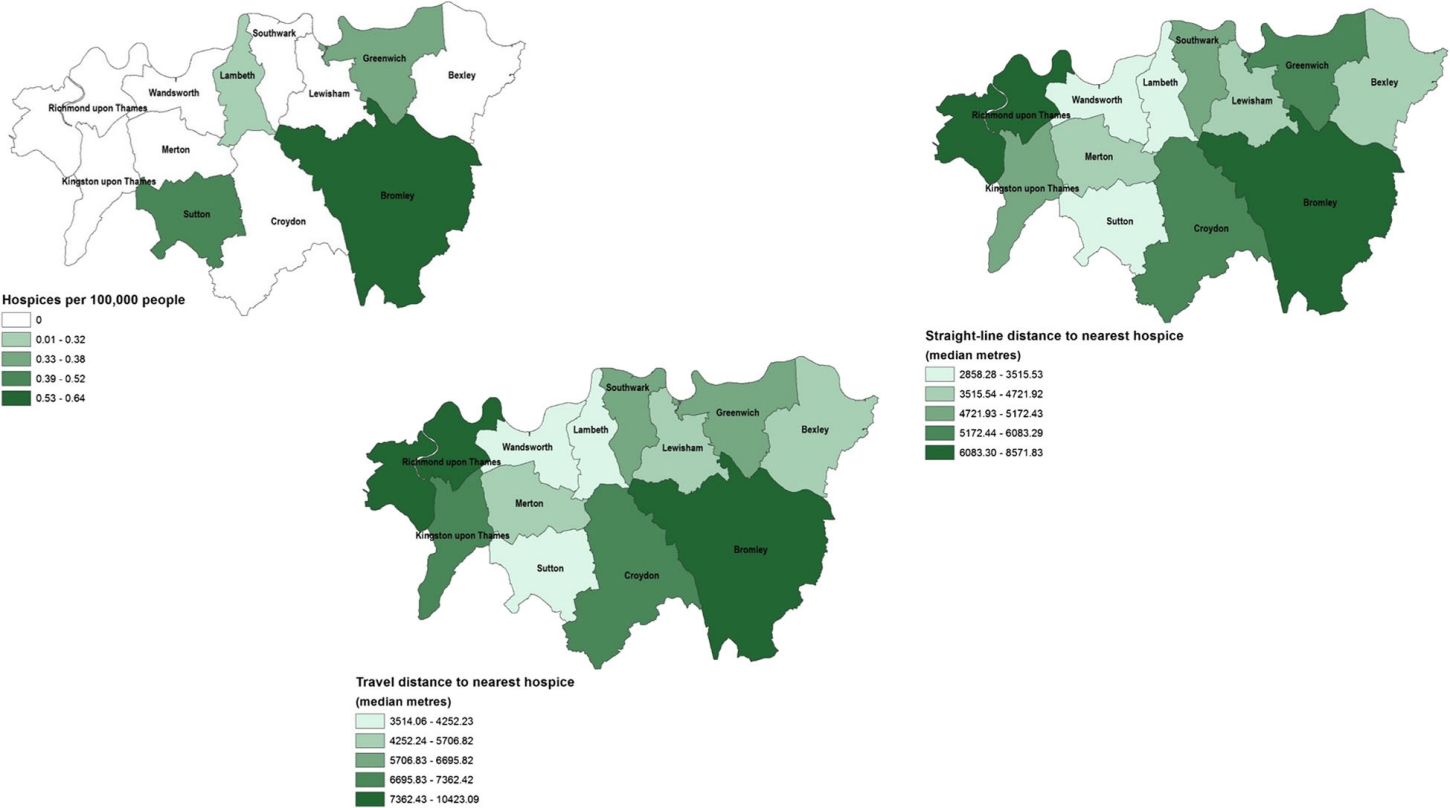
Accessibility measures to the nearest hospice, by borough, 2012. The digital boundary file contains National Statistics data © Crown copyright and database right [2012] and contains Ordnance Survey data © Crown copyright and database right [2012]

Figures 1, 2 and 3 show the ratios of facilities by borough and different distance measures (borough median of straight-line and travel distances) to the nearest hospital, care home and hospice respectively. The maps provide visual recognition of the similarities between the three different distance measurements for healthcare facility type. In addition, the density of care homes and hospitals by borough population (the map in the top left of each figure) appears somewhat inversely related to distance measurements, though this is not as clear for hospices.

The median and range of distances for all decedents to the nearest hospital, care home and hospice using the different methods is shown in Additional file 1: Table S1. The travel distances are longer than the Straight-line distances. Travel times to the different healthcare facilities are inversely related to the number and density of the facilities throughout South London.

The correlation matrix (Table 1) shows the Spearman's correlation coefficients for each of the accessibility measurements by different healthcare facility (hospital, care home and hospice). The two measures of distance (straight-line and travel) are very strongly correlated in all three healthcare facility types; hospital (rho = 0.97), care home (rho = 0.94) and hospice (rho = 0.99). Travel times are also highly correlated with the distance measures, though less strongly than distances (rho range = 0.84–0.95). All correlations are significant at *p* < 0.001.

**Table 1:** Correlations of different measures of geographical accessibility – Spearman's rank correlation coefficients (ρ) (*n* = 18,165)

Hospital (nearest)	Straight-line distance	Travel distance	Travel time
Straight-line distance	1.00		
Travel distance	0.97	1.00	
Travel time	0.84	0.88	1.00
Care home (nearest)	Straight-line distance	Travel distance	Travel time
Straight-line distance	1.00		
Travel distance	0.94	1.00	
Travel time	0.88	0.95	1.00
Hospice (nearest)	Straight-line distance	Travel distance	Travel time
Straight-line distance	1.00		
Travel distance	0.99	1.00	
Travel time	0.93	0.95	1.00

## Discussion

This study is the first to statistically compare geographical accessibility measures to PEoLC-related facilities using individual level death registration data, therefore calculating individual distances from place of residence postcode to healthcare facilities, rather than using an area-level distance. This removes assumptions made using area-level data of homogeneity amongst all people in each area and provides additional statistical significance. The data show that simple methods of calculating geographical accessibility (distances and travel times) were strongly correlated and these correlations were equally high for geographical accessibility from place of residence to the nearest hospital, care home and hospice. Our results are consistent with other comparisons of different healthcare services research, where similarly high correlations between different methods of assigning geographical accessibility were detected [7, 11, 24].

This work is useful to inform decisions on which method to use in future geographical accessibility studies and provides useful guidance in assessing distances for policy making, service planning and resource allocations in the PEoLC-related field, for care providers in assessing the geographical accessibility of their services to patients and families, and for commissioners in planning services, particularly in areas where there might be longer distances to inpatient specialist palliative care services. Our findings reassure PEoLC service providers and interested groups (e.g. GPs) that simple distance measures, which are easier to calculate, are a useful proxy to more complex travel and modelling analysis techniques which may not be available to them. For researchers, it facilitates to bring insights on geographical accessibility to the PEoLC field when GIS expertise are still being developed. In future analyses involving the calculation of geographical accessibility measures (including within the PEoLC field) investigators can have reasonable confidence in a more simple and straightforward way of calculating accessibility (straight-line distance) being just as suitable as more complex and data heavy modelling methods.

A significant strength of this study is the use of individual level data using full postcodes, rather than a small area centroid (e.g. Census Output Area or LSOA). The median number of households per postcode in the UK is 14, which allows for more precision than using a small area such as LSOA, but could still potentially introduce some measurement error. Our work in South London is probably less affected by this error than rural areas, where a single postcode can encompass a far larger geographical area. Repetition of this analysis using data from rural and suburban communities would be informative and could establish if the same conclusions could be drawn or if there are differences in these correlations in different geographical settings.

One of the limitations in this analysis is that more complex measures of calculating geographical accessibility were not included (e.g. floating catchment area modelling). However, previous comparisons have used two [12, 22], three [6, 7] and four [24] different methods in their analyses rather than all possible methods of calculating geographical accessibility. Limitations described in the background for each of the methods chosen for inclusion in this analysis remain valid, including the assumption that people will travel to their nearest facility and that travel along road networks is by an average speed, rather than taking into account conditions which may influence travel times (roadworks, adverse weather conditions, speed limits). In a large metropolitan area such as South London, there is often a choice of healthcare facilities, rather than just one large hospital in the region, and people may not travel to their nearest facility. There is also a well-developed network of SPC services in South London, inpatient and also within the community, which is not captured in this work. The other principle limitation of this analysis, is that it focuses only on South London, with 99.3% of decedents included living in areas classified as urban major conurbation areas by ONS. This does question the generalisability of these findings to other parts of the UK and internationally. Nonetheless, there is geographical variability within South London, more suburban and sparsely populated areas of Bromley and Richmond upon Thames are very different from the inner-city metropolitan urban areas. This analysis, combined with previous comparisons, enables analysts to come to a judgement with reasonable confidence about which method to use for large urban areas. This analysis does not incorporate community and outreach services provided by SPC teams and district nurses as this data is difficult to capture and was not readily available for South London.

## Conclusions

Different methods of calculating geographical accessibility, distance measurements (straight-line and travel) are highly correlated with each other as well as with travel times for decedents in a large metropolitan area with well-developed palliative and end of life care service provision. Therefore, the use of more simple straight-line measurements is useful in assessing geographical accessibility.

## Additional file

Additional file 1: Table S1. Sample characteristics and median distances to nearest hospital, care home and hospice. (DOCX 16 kb)

## Abbreviations

A & E: Accident and Emergency
CQC: Care Quality Commission
HSCIC: Health and Social Care Information Centre
LCA: London Cancer Alliance
LSOA: Lower Super Output Area
NCPC: National Council for Palliative Care
ONS: Office for National Statistics
PEoLC: Palliative and End of Life Care
SPC: Specialist Palliative Care

## References

[1] Penchansky R, Thomas JW (1981). The concept of access: definition and relationship to consumer satisfaction. Med Care.

[2] Bissonnette L, Wilson K, Bell S (2012). Neighbourhoods and potential access to health care: The role of spatial and aspatial factors. Health Place.

[3] Langford M, Higgs G (2006). Measuring potential access to primary healthcare services: the influence of alternative spatial representations of population. Prof Geogr.

[4] Dejardin O, Jones AP, Rachet B (2014). The influence of geographical access to health care and material deprivation on colorectal cancer survival: Evidence from France and England. Health Place.

[5] Martin D, Roderick P, Diamond I (1998). Geographical aspects of the uptake of renal replacement therapy in England. Int J Popul Geogr.

[6] Fone D, Christie S (2006). Comparison of perceived and modelled geographical access to accident and emergency departments: a cross-sectional analysis from the Caerphilly Health and Social Needs Study. Int J Health Geogr.

[7] Haynes R, Jones A, Sauerzapf V (2006). Validation of travel times to hospital estimated by GIS. Int J Health Geogr.

[8] Jordan H, Roderick P, Martin D (2004). Distance, rurality and the need for care: access to health services in South West England. Int J Health Geogr.

[9] Martin D, Wrigley H, Barnett S (2002). Increasing the sophistication of access measurement in a rural healthcare study. Health Place.

[10] Jones SG, Ashby AJ, Momin SR (2010). Spatial implications associated with using Euclidean distance measurements and geographic centroid imputation in health care research. Health Serv Res.

[11] Fortney J, Rost K, Warren J (2000). Comparing alternative methods of measuring geographic access to health services. Health Serv Outcome Res Methodol.

[12] Phibbs C, Luft H (1995). Correlation of travel time on roads versus straight line distance. Med Care Res Rev.

[13] Cinnamon J, Schuurman N, Crooks VA (2008). A method to determine spatial access to specialized palliative care services using GIS. BMC Health Serv Res.

[14] Cinnamon J, Schuurman N, Crooks VA (2009). Assessing the suitability of host communities for secondary palliative care hubs: a location analysis model. Health Place.

[15] Schuurman N, Amram O, Crooks VA (2015). A comparative analysis of potential spatio-temporal access to palliative care services in two Canadian provinces. BMC Health Serv Res.

[16] Currow DC, Allingham S, Bird S (2012). Referral patterns and proximity to palliative care inpatient services by level of socio-economic disadvantage. A national study using spatial analysis. BMC Health Serv Res.

[17] Connor SR, Elwert F, Spence C (2007). Geographic variation in hospice use in the United States in 2002. J Pain Symptom Manag.

[18] Wang S-Y, Aldridge MD, Gross CP (2015). Geographic variation of hospice use patterns at the end of life. J Palliat Med.

[19] Madigan EA, Wiencek CA, Vander Schrier AL (2009). Patterns of community-based end-of-life care in rural areas of the United States. Policy Polit Nurs Pract.

[20] Lindley LC, Edwards SL. Geographic access to hospice care for children with cancer in Tennessee, 2009 to 2011. Am J Hosp Palliat Care. 2015;32(8):849-54. doi:10.1177/1049909114543641. 10.1177/1049909114543641 PMC4294986 25028742

[21] Carlson MD, Bradley EH, Du Q (2010). Geographic access to hospice in the United States. J Palliat Med.

[22] Gatrell AC, Wood DJ (2012). Variation in geographic access to specialist inpatient hospices in England and Wales. Health Place.

[23] Gomes B, Higginson IJ (2006). Factors influencing death at home in terminally ill patients with cancer: systematic review. BMJ.

[24] Apparicio P, Abdelmajid M, Riva M (2008). Comparing alternative approaches to measuring the geographical accessibility of urban health services: Distance types and aggregation-error issues. Int J Health Geogr.

