# Supplementary material for: Measuring geographical accessibility to palliative and end of life (PEoLC) related facilities: a comparative study in an area with well-developed specialist palliative care (SPC) provision
Source: BMC Palliat Care. 2017 Jan 26;16:14. doi: 10.1186/s12904-017-0185-0 (PMC5270238; doi:10.1186/s12904-017-0185-0)
Supplement: Additional file 1: Table S1. — Sample characteristics and median distances to nearest hospital, care home and hospice. (DOCX 16 kb) [file 12904_2017_185_MOESM1_ESM.docx]

Table S1: Sample characteristics and median distances to nearest hospital, care home and hospice

|  | Nearest healthcare facility | | | | | | | | | | | |
| --- | --- | --- | --- | --- | --- | --- | --- | --- | --- | --- | --- | --- |
| **All deaths = 18,165** | Hospital (n=26) | | | | Care home (n=1076) | | | | Hospice (n=5) | | | |
|  | mean | sd | median | range | mean | sd | median | range | mean | sd | median | range |
| Methods |  |  |  |  |  |  |  |  |  |  |  |  |
| Straight-line distances (metres) | 2630.2 | 1323.1 | 2533.4 | 0-7859.8 | 437.9 | 379.5 | 364.6 | 0-3353.6 | 5081.8 | 2597.5 | 4825.5 | 91.2-17173.0 |
| Travel distances (metres) | 3441.5 | 1634.7 | 3309.7 | 0-10070.4 | 687.5 | 574.0 | 593.2 | 0-4736.9 | 6283.8 | 3132.6 | 5909.8 | 101.4-19577.1 |
| Travel times (minutes driving) | 5.3 | 2.2 | 3.6 | 0-19.0 | 1.5 | 1.2 | 1.3 | 0-9.9 | 8.5 | 3.9 | 8.0 | 0.3-28.9 |
